# Raging Hormones: Why Age-Based Etiological Conceptualizations of the Development of Antisocial Behavior Are Insufficient

**DOI:** 10.3389/fnbeh.2022.853697

**Published:** 2022-04-12

**Authors:** Stuart F. White, S. Mariely Estrada Gonzalez, Eibhlis M. Moriarty

**Affiliations:** ^1^Boys Town National Research Hospital, Omaha, NE, United States; ^2^Department of Psychology, University of Texas Rio Grande Valley, Edinburg, TX, United States

**Keywords:** antisocial behavior, puberty, brain, neuroscience, law

## Abstract

Developmental science, particularly developmental neuroscience, has substantially influenced the modern legal system. However, this science has typically failed to consider the role of puberty and pubertal hormones on development when considering antisocial behavior. This review describes major theoretical positions on the developmental neuroscience of antisocial behavior and highlights where basic developmental neuroscience suggests that the role of puberty and pubertal hormones should be considered. The implications of the current state of the science with respect to developmental neuroscience is considered, particularly what is known in light of development beyond puberty. This review shows that development continues to an older age for many youth than the legal system typically acknowledges. The plasticity of the brain that this continued development implies has implications for the outcome of interventions in the legal system in ways that have not been explored. Future directions for both developmental scientists and legal professions are recommended.

## Introduction

The use of biological psychology and neuroscience evidence in the courtroom has expanded dramatically in recent decades (Gazzaniga, 2011; Jones et al., 2014; Farahany, 2015). Developmental science, particularly developmental neuroscience, is having an impact. For example, relatively recent US Supreme Court decisions limiting the type and duration of punishment for crimes committed by adolescents explicitly drew on the developmental psychology literature (Steinberg, 2013). This expanding use of scientific evidence in the courtroom speaks well of both the scientific and legal communities; however, it also raises some concerns (see e.g., Bonnie and Scott, 2013). As the application of science in legal contexts increases, any gaps or weaknesses in the literature carry a greater risk of causing harm *via* misapplication of judicial/legal power.

**FIGURE 1:**
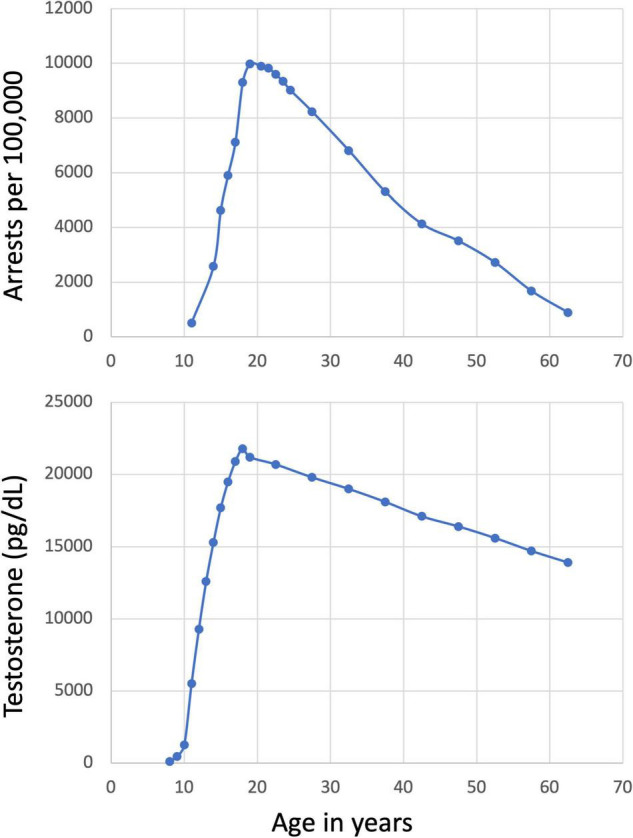
Arrest rates and testosterone levels by age. Increasing arrest rates and increasing levels of testosterone by age are highly correlated (*r* = 0.863, *p* < 0.001). The arrest data shown are from the US in 2014 (Snyder et al., 2019) and the testosterone levels are the maximum of the normal range for males (Forest et al., 1973 as cited by the Mayo Clinic; https://www.mayocliniclabs.com/test-catalog/overview/83686#Clinical-and-Interpretive). The correlation with arrest rates and female testosterone are similarly large (*r* = 0.933, *p* < 0.001).

Issues of development are central to a variety of legal decisions and have potentially enormous consequences for individuals (Steinberg, 2009). Dramatic increases in self-reported antisocial behavior (Moffitt, 2003, 2018) and related official records (e.g., arrest rates; see Figure 1; Snyder et al., 2019) are observed during adolescence. This phenomenon is seen across nations (e.g., Rowland et al., 2019), has been thoroughly documented as a public health problem (e.g., O’Connell et al., 2009), and is of great concern in the criminal justice system (Steinberg, 2009). Thus, it is unsurprising that developmental neuroscience, particularly as it relates to antisocial behavior, is of tremendous interest to the legal system.

Before moving forward, it will be important to define antisocial behavior. Antisocial behavior is behavior that violates the basic rights of others, including emotional or physical harm (Calkins and Keane, 2009). To date, the majority of the developmental neuroscience seeking to explain antisocial behavior has focused on age, but has not adequately considered other aspects of development, including puberty. Even more critically, the literature has inadequately considered the hormones that drive developmental change during and after puberty. This is somewhat surprising given that (i) age and puberty differentially impact the brain and behavior (Crone and Elzinga, 2015); (ii) increased antisocial behavior has been observed as a function of both pubertal status (Hemphill et al., 2010) and pubertal timing (see Negriff and Susman, 2011), and (iii) peak age for antisocial behavior at approximately 19 years (Snyder et al., 2019), several years after youth are “adult-like” in terms of size and general appearance (Dorn et al., 2006). We argue that a closer examination of the role of hormones in development (i.e., during and after puberty) on risk for antisocial behavior is critical to ensure that the science used by the courts is accurate and complete as possible. In this review, we will briefly describe pubertal and brain development generally. We will then highlight the neurobiology underpinning some major theoretical perspectives on antisocial behavior and discuss what is known regarding pubertal development and implicated biological systems. The relative lack of studies directly examining pubertal development and the neurobiology of antisocial behavior directly will be noted. Finally, the implications of these data to the legal system will be discussed and recommendations for future work will be made.

## Brain Development During Adolescence

### Puberty

Puberty is a critical period of development that largely coincides with early to mid-adolescence, where hormone-driven processes facilitate sexual maturation (Grumbach and Styne, 2003). Two broad hormonal processes drive pubertal development. Puberty begins with adrenarche, which is when the adrenal gland releases increased amounts of androgens, including dehydroepiandrosterone (DHEA), dehydroepiandrosterone sulfate (DHEAS), and androstenedione (Palmert et al., 2001; Biro et al., 2014). This androgen release leads to the development of some secondary sex characteristics, including pubic hair, acne, and body odor (Grumbach and Styne, 2003). Adrenarche typically occurs between 6 and 9 years (earlier in girls than boys) and levels of androgens increase until the early 20’s (Figure 1; Havelock et al., 2004). Gonadarche typically begins between 9 and 14 in females and 10 and 15 in males (Dorn et al., 2006) and involves increased production of testosterone from the testes in males and estradiol from the ovaries in females (Grumbach and Styne, 2003). Gonadal hormones are involved in the development of primary and secondary sex characteristics, including genital and (in females) breast development, body/facial hair growth, and the growth spurts associated with puberty (Grumbach and Styne, 2003). Both gonadal and adrenal hormones are involved in pubertal brain development (Herting and Sowell, 2017). Variation in the timing of the initiation of puberty is not well understood, but is thought to be influenced by levels of body fat, hypothalamic-pituitary-adrenal (HPA) axis activity, and genetics (Cizza et al., 2001). Notably, there is some indication that pubertal development varies by race/ethnicity (Keenan et al., 2014; Deardorff et al., 2021), though it is unclear whether these differences are actually race/ethnicity-based or due to factors correlated with, but not attributable to race/ethnicity (e.g., income, Deardorff et al., 2019).

### Brain Development During Adolescence

Thorough reviews of adolescent brain development can be found elsewhere (e.g., Casey et al., 2008; Blakemore, 2012; Konrad et al., 2013). Briefly, major changes in brain structure take place between childhood and adulthood (Giedd et al., 1999). Structurally, the “pruning” of synapses (Huttenlocher and Dabholkar, 1997) and thinning of gray matter are observed (Gogtay et al., 2004), along with *increases* in white matter (Gogtay et al., 2004; Perrin et al., 2008). Moreover, substantial changes in neurotransmitter systems are also occurring (Murrin et al., 2007). These structural and organizational changes are thought to contribute to corresponding changes in brain function. Briefly, as adolescence progresses, the prefrontal cortex exerts more control over sensory and affective regions of the brain, temporally consistent with the progression of structural changes in the brain (Casey et al., 2010). These changes in brain structure and function are linked with behavioral changes seen during adolescence (Casey et al., 2010), including increased responses to reward (Galván, 2013) and greater risk-taking (Galvan et al., 2007). Critically, the majority of studies examining brain development have considered brain development primarily as a function of age. This is despite differential impacts of age and puberty on both brain and behavior (Crone and Elzinga, 2015) and the roughly 5-year range of normal pubertal onset (Dorn et al., 2006). It is perfectly normal to have one 14 year-old in Tanner stage one (pre-puberty) and another in Tanner stage 5 (“adult-like”); treating these youth as equivalent neurobiologically is unjustified.

### Brain Development and Puberty

While increases in hormones begin during puberty, both adrenal and gonadal hormones continue to increase into the late teens/early 20’s (Havelock et al., 2004; Kelsey et al., 2014), after most individuals are Tanner Stage 5 and “adult-like” (Dorn et al., 2006). These hormone increases appear to roughly parallel the temporal course of brain development (i.e., Gogtay et al., 2004), suggesting at least partially distinct roles in physical vs. neural development. There has been some work specifically looking at the role of puberty in adolescent brain development (for reviews see Blakemore et al., 2010; Herting and Sowell, 2017; Vijayakumar et al., 2018). Briefly, as puberty advances, there are decreases in gray matter volume and increases in white matter similar to what is seen in the age literature (Vijayakumar et al., 2018). When the brain development literature has considered puberty, it has tended to consider secondary sex characteristics (e.g., Tanner staging) as the marker of pubertal development (see Vijayakumar et al., 2018). However, this approach has limitations. As reviewed by Dorn et al. (2006), there are reliability and validity problems with both self-report and physician-exam measures of secondary sex characteristics. Furthermore, hormones are thought to drive both the development of secondary sex characteristics and brain development (Grumbach and Styne, 2003). While more work is needed, a number of studies have shown that changes in pubertal hormones are associated with structural changes in the brain in humans (e.g., Nguyen et al., 2013, 2017; Nguyen, 2018). Indeed, structural neuroimaging and hormone work has found that adrenal hormones may impact brain development more during earlier stages of puberty, while gonadal hormones may influence brain development primarily later in puberty (Herting et al., 2017). With respect to *functional* neuroimaging, far less work has been conducted and the results have been less consistent (see Vijayakumar et al., 2018). As noted by Vijayakumar et al. (2018), this is likely a function of the limited work that has been conducted, the variability in behavioral paradigms completed during scanning, and the differing methods of examining pubertal development.

## The Neurobiology of Antisocial Behavior and Developmental Considerations

In the following sections, the neurobiology of antisocial behavior will be considered using two approaches. The first approach is to consider typical neurobiological changes observed during adolescent development. As noted above, antisocial behavior, behavior that violates the rights of other, including physical and emotional harm, generally increases over the course of adolescence. Theoretical positions postulating the role of typical developmental neurobiological changes in increasing the risk for antisocial behavior will be discussed. Following this, what is known about developmental changes during adolescence in theoretically relevant neurobiological systems will be reviewed.

The second approach is to consider the neurobiology of psychiatric disorders that are associated with an increased risk of antisocial behavior and have a high prevalence in adolescence. The neurobiology of Disruptive Behavior Disorders, including Conduct Disorder and Oppositional Defiant Disorder, which are common in adolescence, will be discussed. Several theoretical positions on the neurobiology of Disruptive Behavior Disorders have been detailed in the literature (e.g., Matthys et al., 2013; Rosell and Siever, 2015; Blair et al., 2016). Unsurprisingly, there is variation in the specifics of these theoretical positions. However, there are common cognitive processes, and associated neural systems, which are broadly implicated in antisocial behavior, including reduced response to reward information and dysfunction in avoidance learning (see Table 1). Moreover, reduced response to fear/distress cues, associated with callous-unemotional traits, and increased threat/emotional reactivity, associated with trauma exposure, have both been linked to antisocial behavior. Changes in these systems associated with puberty will be reviewed.

**TABLE 1:** Neurobiological systems implicated in antisocial behavior.

Cognitive process	Neural regions	Hormones implicated	Proposed mechanism
Emotion regulation/Behavioral control	**Sub-cortical:** striatum, amygdala **Cortical:** dorsolateral prefrontal cortex, orbitofrontal cortex/ventromedial prefrontal cortex	Testosterone, DHEA	In typical development, regulation of sub-cortical regions by cortical regions is less effective, which leads to poor emotion regulation and behavioral control. Testosterone and DHEA may influence the connectivity between cortical and sub-cortical regions.
Reward response	Ventral striatum	Testosterone	Failure to properly encode reward information may interfere with decision-making and lead to increased risk-taking/sensation-seeking and/or frustration. Testosterone is associated with increased ventral striatum response to reward, though not in all studies.
Avoidance learning	Anterior insula cortex	Estradiol (in females), DHEA	Activation in anterior insula cortex is part of a behavior change response. Failure to generate this response is associated in increased levels of antisocial behavior. Increased estradiol and DHEA have been associated with disrupted anterior insula cortex functioning.
Emotional empathy	Amygdala	Testosterone-cortisol coupling	Failure to represent the fear/distress of others in the amygdala reduces the aversive nature of fear/distress cues in others, is associated with callous-unemotional traits and increases the likelihood of antisocial behavior. Disrupted testosterone-cortisol coupling is associated with callous-unemotional traits.
Threat response	Amygdala	Testosterone	Increased amygdala responsiveness to threat cues in the environment makes a reactive aggression response more likely. Testosterone is associated with greater amygdala response to threat cues.

*Several important neurobiological systems are implicated in antisocial behavior across major theoretical perspectives and show substantial change during adolescence.*

### Typical Adolescent Development and Increased Risk for Antisocial Behavior

There are two main biologically oriented theorical perspectives on why there is a dramatic increase in antisocial behavior in adolescence. The first perspective postulates that increased antisocial behavior can be attributed to uneven maturation of different brain regions (Cohen and Casey, 2014; Jones et al., 2014). The second perspective, largely drawn from the animal literature (e.g., Berthold and Quiring, 1944), implicates increases in testosterone with increases in aggression, particularly in males. Notably in a human neuroimaging study, testosterone was found to modulate the relationship between amygdala and prefrontal cortex development and this relationship between amygdala and prefrontal cortex development was, in turn, associated with aggression (Nguyen et al., 2016). Despite these findings, the link between testerosterone and aggression, particularly in the human literature, however, is not a straightforward one.

#### Brain Development in Adolescence: Neurobiological Theory of Antisocial Behavior

It has been argued that increased antisocial behavior associated with adolescence can be attributed to uneven maturation of different brain regions (Cohen and Casey, 2014; Jones et al., 2014). The amygdala is important for emotion processing, including response to threats (LeDoux, 2012), and the striatum is critical for reward processing (O’Doherty et al., 2017). Both are regions implicated in antisocial behavior (Matthys et al., 2013; Cohen and Casey, 2014; Blair et al., 2016). Phylogenetically older regions, including the amygdala and the striatum, are thought to mature earlier and more quickly than cortical regions that regulate them (Casey et al., 2008; Steinberg, 2008), including regions of dorsolateral prefrontal cortex (Cohen et al., 2016) and ventromedial/orbitofrontal cortex (Breiner et al., 2018). This increased sub-cortical activation has been implicated in poorer cognitive control of both behavior and emotion in adolescence relative to adulthood (Breiner et al., 2018). In the original iterations of the model, this imbalance was used to explain adolescent risk-taking. Poorer cognitive control of emotion and behavior increase risk-taking behavior, which in turn is argued to increase the risk of antisocial behavior (Steinberg, 2009; Cohen and Casey, 2014). Correspondingly, as brain development continues into the early 20’s, levels of antisocial behavior in the population drop correspondingly (Steinberg, 2009).

#### Brain Development in Adolescence: Developmental Changes in Neurobiology

As previously noted, age and puberty have differential developmental impacts on the brain (Crone and Elzinga, 2015). Substantial developmental changes in the modulatory relationship between regions of cortex and sub-cortical structures occur in adolescence (Casey et al., 2008; Breiner et al., 2018). With respect to pubertal changes, increasing testosterone is associated with reduced functional connectivity between ventral medial prefrontal cortex (vmPFC) and amygdala (Peters et al., 2015; Spielberg et al., 2015). Similarly, increasing dehydroepiandrosterone (DHEA) is associated with reduced functional connectivity between insula and amygdala (Barendse et al., 2018). Differences in connectivity between amygdala and cortical regions/anterior insula are thought to reflect changes in how emotional/motivational information from the amygdala is incorporated into decision-making and response selection (e.g., Blair and Cipolotti, 2000). However, increasing *age* during adolescence is associated with *increased* resting state functional connectivity between vmPFC and amygdala (Gabard-Durnam et al., 2014). There is less data with respect to the striatum, although recent developmental neuroimaging work suggests that vmPFC-amygdala connectivity develops over the course of adolescence and is a pre-requisite for successful regulation of the striatum during behavior (see Casey et al., 2019). This position posits that the developmental changes observed in the functional connectivity between vmPFC and amygdala have a direct impact on the ability of the adolescent brain to regulate the striatum at different developmental stages. In short, there is evidence that changes in hormones during puberty are associated with changes in neural processes that are implicated in antisocial behavior. However, work in this area is limited.

#### Hormone Changes in Adolescence: Neurobiological Theory of Antisocial Behavior

The idea that there is a relationship between hormones and aggression, particularly testosterone, is an old one (see Sapolsky, 1997). Focusing on the human literature, a positive association between testosterone and aggression levels have been found in meta-analytic work (Geniole et al., 2019) and, as noted above, the observed increase in adolescent antisocial behavior coincides with the beginning of increases in testosterone levels associated with puberty. However, the effect size of the association between testosterone and antisocial behavior is small and restricted to males (Geniole et al., 2019). It has been proposed that testosterone, at least in humans, has a greater influence on social dominance rather than directly on antisocial behavior (e.g., Mazur and Booth, 1998). Moreover, there is evidence to suggest that testosterone is only associated with social dominance when the testosterone to cortisol ratio is high (Mehta and Josephs, 2010; Mehta et al., 2015; Grotzinger et al., 2018), though this has not always been reported (for a review see Grebe et al., 2019).

Additionally, there is evidence to suggest that increased levels of adrenal androgens (e.g., DHEA) are associated with antisocial behavior (van Goozen et al., 1998; Dorn et al., 2009). However, similar to testosterone, these findings are also inconsistent (e.g., Shenk et al., 2012). Hormones have typically been examined one at a time, but increasingly, as noted with testosterone and cortisol above, the interaction between endocrine systems is implicated in influencing the likelihood of any specific behavior (e.g., Mehta and Josephs, 2010; Marceau et al., 2015). It is therefore quite possible that the association between adrenal hormones and antisocial behavior is complex and involves multiple endocrine systems. In sum, there is evidence that hormones play a role in modulating antisocial behavior, but that role remains unclear (Carré and Archer, 2018).

#### Hormone Changes in Adolescence: Developmental Changes in Neurobiology

As previously noted, dramatic increases in numerous hormones occur during puberty, including DHEA, testosterone, cortisol and estradiol (Grumbach and Styne, 2003; Netherton et al., 2004). Moreover, males show much greater increases in testosterone levels relative to females (Grumbach and Styne, 2003). Both of these findings are consistent with increases in antisocial behavior seen in adolescence (Moffitt, 2018), as well as the gender gap in antisocial behavior (Russell et al., 2014). Importantly, however, the gender gap in antisocial behavior is actually lowest during adolescence (Russell et al., 2014) when sex differences in hormone levels are dramatically increasing (Grumbach and Styne, 2003).

The relationship between increasing pubertal hormonal levels, notably testosterone, and antisocial behavior may be only correlational and no direct, causal relationship may exist. It has been previously observed that there are complex, bidirectional relationships between various levels of biological functioning (e.g., gene expression and cellular, endocrine, and neural functioning; Gottlieb, 2007). As hormones influence behavior *via* modulation of neural systems that directly control behavior (Bogdan et al., 2012), integrating neuroimaging and neuroendocrine work will be needed to understand the relationship between hormones and antisocial behavior. In line with this idea, it has been argued that the role of hormones, specifically testosterone and cortisol, in influencing antisocial behavior can best be understood in terms of how these hormones modulate the functional connectivity between amygdala and prefrontal cortex (Rosell and Siever, 2015). As described above, this cortical-subcortical connectivity is implicated in increasing risk for antisocial behavior in the developmental neuroimaging literature (Cohen and Casey, 2014).

The relationship between hormones and other social behaviors that are indirectly associated with antisocial behavior may help explain the significant, but limited, association between aggression and testosterone. For example, it has been argued that testosterone’s influence is primarily on social dominance (e.g., enhancing social status, winning competitions), not directly on antisocial behavior (Mazur and Booth, 1998). Additionally, increases in testosterone have been associated with increases in risk-taking (see Apicella et al., 2015). Increases in risk-taking are observed in adolescence (Galvan et al., 2007) and are correlated with antisocial behavior (Steinberg, 2008). Thus, while continued investigations of the role of hormones in altering risk for antisocial behavior is warranted, simple correlational analyses of hormone levels with rates of antisocial behavior are likely to be insufficient.

## Neurobiological Dysfunction in Psychopathology Associated With Antisocial Behavior

There are a number of neurobiological systems theoretically implicated in Disruptive Behavior Disorders (DBDs). DBDs include Conduct Disorder and Oppositional Defiant Disorder, both of which are associated with an increased risk of antisocial behavior. In this section, the systems most consistently implicated in the development of Disruptive Behavior Disorders in the literature will be reviewed.

### Reduced Response to Reward: Neurobiological Theory of Antisocial Behavior

Abnormal response to reward information has been observed in antisocial youth (e.g., Frick and White, 2008; Frick et al., 2014; Blair et al., 2016). Failure to adequately encode reward information may increase the risk for antisocial behavior. One model focuses on the link between reward processing, frustration, and reactive aggression (Blair, 2010). The accurate encoding of reward information is necessary to accurately predict likely outcomes following actions in a given environment. Disrupted representation of reward information will result in poor prediction of likely outcomes in the environment leading to increased frustration. Frustration is associated with greater risk of reactive aggression (Blair, 2010). Alternatively, inaccurate representation of reward information might lead to increased risk- and sensation-seeking behavior, which is also associated with antisocial activities (Mann et al., 2017).

Neurobiological models of antisocial behavior implicate dysfunction within the striatum during reward processing (Matthys et al., 2013; Rosell and Siever, 2015; Blair et al., 2016). The striatum is a key region in the reward system and is responsive to reward cues and rewarding feedback (O’Doherty et al., 2006). Several studies have shown reduced activation within striatum to reward in antisocial youth (Finger et al., 2011; White et al., 2013; Cohn et al., 2015; Holz et al., 2017), though at least one study reported increased reward response (Bjork et al., 2010). Several studies have also reported reduced activation to reward in regions of vmPFC/orbitofrontal cortex (Rubia et al., 2009; Finger et al., 2011; White et al., 2013; Schwenck et al., 2017), which are implicated in reward value representation and/or in the regulation of striatum (see e.g., O’Doherty et al., 2017). Multiple theoretical positions associate striatal dysfunction during reward processing with the decision-making problems associated with increased risk for antisocial behavior (e.g., Matthys et al., 2013; Rosell and Siever, 2015; Blair et al., 2016). Supporting these theoretical positions, two Activation Likelihood Estimate (ALE) meta-analyses implicate dysfunction in striatum and interconnected regions of cortex in antisocial behavior in adolescents (Alegria et al., 2016; Noordermeer et al., 2016).

### Reduced Response to Reward: Developmental Changes in Neurobiology

A substantial amount of change occurs in the striatum during pubertal development. Structurally, an accelerated longitudinal study found that striatum volume was negatively associated with pubertal development (Goddings et al., 2014). Increasing testosterone occurring as a result of puberty has been associated with increased striatal response to threat cues (Spielberg et al., 2014). Moreover, longitudinal studies have shown that striatal response to reward information rises from early adolescence to a peak in mid-adolescence before declining (Braams et al., 2015; Vijayakumar et al., 2019), which is consistent with cross-sectional data (Casey et al., 2008). Notably, level of reward response in striatum has been associated with substance abuse, an antisocial behavior (Braams et al., 2016). Interestingly, other evidence suggests a more complex relationship between pubertal hormones and reward. White et al. (2020) found that increased testosterone reactivity is associated with reduced striatum and vmPFC activation in rewarded relative to unrewarded trials. In the same sample, the gonadal hormone estradiol was also associated with reduced striatal response to rewarded relative to unrewarded trials in females (Ladouceur et al., 2019). Importantly, in the paradigm considered in both studies, reward information was irrelevant to successful task completion. White et al. (2020) argued that this finding is consistent with hormones being highly context-dependent, consistent with theoretical positions (e.g., Roney, 2016).

### Dysfunctional Avoidance Learning: Neurobiological Theory of Antisocial Behavior

Poor decision-making is associated with antisocial behavior (for reviews see Matthys et al., 2013; Rosell and Siever, 2015; Blair et al., 2016). In recent years, the use of computational neuroimaging has begun to better illustrate the regions of the brain involved in specific computations involved in decision-making relative to earlier neuroimaging work (O’Doherty et al., 2007). One of the essential computations necessary for decision-making is the expected value that becomes associated with an action or stimulus based on previous learning (O’Doherty, 2004). Building on previous work showing increased activation in anterior insula to cues signaling risk or avoidance (Kuhnen and Knutson, 2005; Liu et al., 2007), anterior insula has been shown to represent expected value during avoidance behavior (Rigoli et al., 2016). It has been argued that this representation of expected value in anterior insula is involved in response selection and behavior change (Casey et al., 2001; Budhani et al., 2007; White et al., 2014). Youth with DBDs show reduced representation of expected value in anterior insula when avoiding sub-optimal choices (White et al., 2013, 2014). Notably, the degree of impairment in the representation of expected value during the avoidance of sub-optimal choice has been associated with increased levels of antisocial behavior (White et al., 2016). Failure to appropriately represent the computations needed for successful behavior change might account for the punishment insensitivity observed in antisocial youth (Frick and White, 2008; Matthys et al., 2013; Frick et al., 2014; Rosell and Siever, 2015; Blair et al., 2016; White et al., 2016).

### Dysfunctional Avoidance Learning: Developmental Changes in Neurobiology

To our knowledge, no developmental neuroscience work has specifically considered pubertally driven changes in avoidance learning. However, the anterior insula has been considered in broader developmental neuroscience work on reward response. For example, a meta-analysis of reward processing found that adolescents show greater anterior insula activation during reward processing than adults (Silverman et al., 2015), suggesting adolescent changes in anterior insula functioning. Increasing DHEA during late childhood is associated with reduced functional connectivity between insula and amygdala (Barendse et al., 2018). An additional study found that increasing levels of estradiol in adolescent females were associated with anterior insula activation during social, but not monetary, feedback (Op de Macks et al., 2017). Estradiol is a major driver of pubertal development in females and DHEA is largely responsible for adrenarcheal maturation. These findings point to the possibility that puberty is altering functioning in anterior insula and highlight this as a potentially fruitful target for future work in this area.

### Dysfunctional Amygdala Reactivity: Neurobiological Theory of Antisocial Behavior

With respect to amygdala functioning, there is heterogeneity between antisocial youth in terms of neural correlates. A lack of emotional empathy is specifically associated with the presence of DBDs and callous-unemotional (CU) traits (Viding et al., 2014; Blair et al., 2016). CU traits include a lack of guilt or remorse, a lack of concern about poor performance, callousness, and shallow affect (Frick and White, 2008; Frick et al., 2014) and are included as the “Limited Prosocial Emotions” specifier for Conduct Disorder in DSM-5 (American Psychiatric Association, 2013). The lack of empathy shown by these youth is thought to increase the likelihood of an individual selecting antisocial, particularly aggressive, social tactics (Pardini, 2006; Frick and White, 2008; Blair et al., 2014; Frick et al., 2014). The lack of empathy seen in antisocial youth displaying high levels of CU traits is thought to stem from reduced amygdala response to fear/distress cues in others (Blair et al., 2014, 2016; Viding et al., 2014). Notably, the relationship between CU traits and proactive aggression appears to be mediated by the level of amygdala response to fear/distress cues (Lozier et al., 2014). Furthermore, there is some evidence that CU traits may be related to differences in the relative coupling of the diurnal rhythms of testosterone and cortisol (Johnson et al., 2014).

In contrast, many youth with DBDs not exhibiting CU traits show *increased* amygdala response to fear/threat cues (Viding et al., 2012; Blair et al., 2014, 2016), similar to youth with exposure to traumatic events and maltreatment (McCrory et al., 2017). Indeed, there is a high comorbidity between trauma exposure and antisocial behavior (Lewis et al., 2019). Increased activation of the basic threat system, of which the amygdala is a part (Nelson and Trainor, 2007), is thought to increase the likelihood of increased reactive aggression and other reactive antisocial behaviors (Blair et al., 2016). Notably, even in youth with high levels of CU traits, high levels of trauma are associated with increased, rather than decreased, amygdala response to fearful faces (Meffert et al., 2018). It has been argued that the tendency among antisocial youth to misinterpret ambiguous stimuli as threatening (Crick and Dodge, 1996) can be accounted for by this increased response to fear/threat stimuli (Crowe and Blair, 2008).

### Dysfunctional Amygdala Reactivity: Developmental Changes in Neurobiology

Both elevated and diminished amygdala responsiveness is associated with increased risk of antisocial behavior. The amygdala increases in volume over the course of adolescence, both as a function of age and puberty (Goddings et al., 2014). Longitudinal increases in testosterone as a result of pubertal development are associated with increased amygdala responses to threat cues (Spielberg et al., 2014). In a different longitudinal sample, amygdala responsiveness to faces increased from age 10 to 13 (Moore et al., 2012). Moreover, earlier pubertal timing was associated with increased amygdala reactivity at age 10, with a significant increase in magnitude at age 13 (Moore et al., 2012). Adding data from additional waves of collection, Vijayakumar et al. (2019) found that amygdala responsiveness to emotional expressions increased with pubertal development, peaking at Tanner Stage 3 and then declining over Tanner Stages 4 and 5. Interestingly, in the same study, amygdala responsiveness, at least in females, was found to have an inverse U-shaped relationship with testosterone levels (Vijayakumar et al., 2019). In short, it seems clear that amygdala responsiveness changes occur as a result of puberty, potentially moderated by sex, though further work is needed.

### Hormonal Changes Associated With Disruptive Behavior Disorders: Neurobiological Theory of Antisocial Behavior

Based on the animal literature, a link between hormones, particularly testosterone, and antisocial behavior, especially aggression, has been hypothesized. Indeed, higher levels of testosterone have been found in individuals meeting criteria for DBDs (Aromäki et al., 1999; Chance et al., 2000; Pajer et al., 2006) and in incarcerated individuals (Kreuz and Rose, 1972; Bain et al., 1987; Dabbs et al., 1987; Stålenheim et al., 1998; Maras et al., 2003; van Bokhoven et al., 2006). Importantly, however, evidence suggests that the relationship between testosterone and antisocial behavior in youth with DBDs is moderated by levels of CU traits (Blanchard and Centifanti, 2017), social dominance (Rowe et al., 2004), or by harsh parenting (Chen et al., 2018). Moreover, there is a report of altered coupling between HPG and HPA axes in youth with Conduct Disorder, though only in those who also had high levels of CU traits (Johnson et al., 2014). In sum, while testosterone does appear to be elevated in individuals at high-risk for antisocial behavior, the mechanism by which this association is conveyed remains unclear and is likely moderated by psychosocial context, other hormone systems, and other risk factors. Moreover, as discussed above, the relationship between hormones and antisocial behavior is likely to be mediated by other complex social processes and contextual factors.

### Hormonal Changes Associated With Disruptive Behavior Disorders: Developmental Changes in Neurobiology

As noted above, increases in hormonal output occur during puberty (Grumbach and Styne, 2003). If increased levels of hormones are associated with antisocial behavior in individuals with DBDs, it would make sense that levels of antisocial behavior would increase during puberty. There is some evidence that there is a large spike in antisocial behavior during adolescence in some youth (Moffitt, 2003, 2018; Snyder et al., 2019). However, the peak of antisocial behavior is approximately 19 years (Snyder et al., 2019), which is after puberty typically ends (Dorn et al., 2006), but before hormones have finished increasing (Havelock et al., 2004; Kelsey et al., 2014). Furthermore, in the most consistently, severely, and chronically antisocial youths, antisocial behavior begins before puberty (see Frick, 2012). In short, there is a shortage of data that clearly links hormonal changes associated with puberty to an increased risk of antisocial behavior. A better understanding of the specific biological mechanisms linking hormonal changes and antisocial behavior is needed.

## Summary of the Neurobiology of Antisocial Behavior and Development

The existing literature with respect to antisocial behavior is remarkable in terms of both the clear importance of adolescence to the development of antisocial behavior (Table 1) and the lack of attention to hormone-driven mechanisms that might influence the development of antisocial behavior. The advent of functional neuroimaging has identified at least some neural systems that are implicated in antisocial behavior (e.g., anterior insula and avoidance learning; amygdala and threat response/empathy). Moreover, there has been some work on the developmental neuroscience of antisocial behavior. However, this work has focused mainly on age. The field requires much more work that integrates the existing findings and disentangles age from pubertal development, as well as longitudinal work, allowing for the examination of developmental processes. Finally, there is a pressing need to integrate the hormone, behavior, and the neuroimaging literature in at least two ways. First, because hormones do not directly influence behavior, but instead modulate the neural systems that control behavior (Bogdan et al., 2012), designing experiments that directly measure data at all three levels will provide a much clearer picture of the factors driving antisocial behavior. Second, given the role that hormones play in brain development, both in puberty and beyond, hormones may be a better metric for development than measures of primary and secondary sex characteristics. Work that informs a comprehensive developmental picture of the development of antisocial behavior will need to be conducted to provide various stakeholders with the best possible data.

## Implications for the Legal System

As shown above, there is relatively little known about the role of puberty and pubertal hormones in the development and maintenance of antisocial behavior. However, it is also clear that both pubertal and adolescent development is substantially altering the neural systems that, when dysfunctional, are implicated in increasing risk for antisocial behavior. Moreover, it is also clear that physical and neural development, while related, are not perfectly aligned. With the ever-increasing influence of developmental neuroscience in the legal system, this lack of data has implications in several important areas of legal decision-making.

### Adult Treatment of Juveniles in the Justice System

The legal system has long considered development as a key factor in determining culpability and what appropriate legal dispositions following adjudication should be. For over 100 years, the United States has implemented various juvenile justice systems with the intention of developmentally appropriate legal recourse for juvenile offenders (Bartol, 2002). Notably, while the developmental science of the past several decades has indicated that brain development is not complete until the early- to mid-20s (Gogtay et al., 2004; Cohen et al., 2016), the justice system (in some jurisdictions) has lowered the age at which juveniles can be tried as adults to as low as 13 years (Griffin et al., 2011). The legal system’s approach cannot be justified on the basis of developmental science.

The conflict between the developmental science and the legal system has been noted elsewhere (e.g., Steinberg, 2009; Cohen and Casey, 2014). Developmental scientists have recommended that juveniles 15 years old and younger should *not* be treated as adults by the justice system and that the maturity of older youth be considered on a case-by-case basis when considering transfer to adult court (e.g., Steinberg, 2009). Notably, the studies informing this position have considered age, but not the role of puberty and pubertal hormones. Furthermore, the developmental science would suggest that, as development continues into the early- to mid-20’s, more consideration of the developmental status of individuals in their 20’s should take place in the justice system. For immature individuals in their early 20’s, the more treatment-oriented accountability options of the juvenile justice system may be more appropriate than the adult justice system.

### Reducing Reliance on Age in the Legal System

The onset of puberty has a normative range of approximately 5 years (Dorn et al., 2006). As such, it is normal for there to be substantial differences in levels of pubertal development between individuals, particularly in the early teenage years. The range of height and physical development seen in any junior high school classroom is a testament to this. Despite this normal reality, legal statutes and guidance typically invoke only age (e.g., age of majority, to drive, buy alcohol, etc.). As noted above, there are some youth that are capable of making adult-like decisions in their mid-teens, but this is not ubiquitous (Steinberg, 2009). Considering development more holistically, using behavioral markers, and potentially endocrine markers, would allow the legal system to operate in a fashion more consistent with developmental science.

Importantly, however, a substantial amount of additional scientific work is needed before developmental science can provide adequately validated behavioral or endocrinological markers. A well-validated measure of development/maturity using behavioral markers seems conceivably within reach with sufficient investment, given the research scales current in use (e.g., the Psychosocial Maturity Index; Cauffman and Steinberg, 2000). The gap between research tools and validated instruments ready for use in clinical or legal contexts is immense, however, and substantial work is needed. Endocrinological markers are further away than behavioral markers. Extensive work is still needed to establish reference norms for children and adolescents and to deal with lingering methodological issues with respect hormone assays (e.g., Granger et al., 2004). Moreover, the convergent validity between the behavioral and endocrinological markers of development/maturity will need to be established.

### Neural Plasticity Continues Into Early Adulthood

Adolescence is a time of great change, which conveys the opportunity for extensive change, both positive and negative (Dahl, 2004). The goals of the juvenile justice system are more weighted toward treatment and rehabilitation relative to the adult justice system, with the idea that youth are more malleable than adults (Bartol, 2002). The brain is relatively plastic and changing until the early- to mid-20s (Gogtay et al., 2004; Cohen et al., 2016), which suggests that malleability is present until a later age than currently assumed. Increasing the effort placed on treatment in individuals who were previously not considered treatable may have a positive influence on outcomes. The converse may also be true. By treating 18-year-olds as adults, despite the fact that they are not likely finished maturing, the known detrimental effects of incarceration (e.g., higher levels of depression, substance abuse, reduced life satisfaction; Yi et al., 2017) may be exacerbated.

### Interaction Between Pubertal Development and Systematic Influences on the Justice System

Issues of development in the justice system cannot be considered in a vacuum; individual differences in development interact with other factors that influence the justice system. For example, systemic racism continues to plague the American justice system (Tonry, 2011). Early onset of puberty is associated with increased likelihood of antisocial behaviors throughout adolescence, particularly low-level offenses such as truancy, substance abuse, vandalism, etc. (for a review see Mendle and Ferrero, 2012). African-American youth are rated as less “innocent” and are viewed as older and less “child-like” than same-age White youth (Goff et al., 2014). Critically, police officers over-estimated the age of both African–American and Hispanic youth, suggesting that this bias exists within law-enforcement (Goff et al., 2014). African-American and Hispanic youth, particularly males, are at increased risk for arrest relative to European-American youth (Kirk, 2008; Brame et al., 2012). The interaction between early puberty and being African-American and/or Hispanic on risk for justice-involvement has not been examined explicitly to our knowledge. However, it seems likely that the risk of justice-involvement in early developing African-American and Hispanic youth will be even higher than the risk of justice-involvement for early developing White youth. This interaction is just one example where the justice system must better integrate developmental science into legal practice and understand the interactions between development and societal issues that also influence the justice system.

## Future Directions

There are clearly a number of gaps in the existing literature. However, neuroscience, psychology, and affiliated disciplines have a number of tools that are readily applicable to address these shortcomings. In this final section, we will describe some possible methods for improving the current state of the literature with respect to antisocial behavior and pubertal development.

First, and most critically, the field needs to more regularly consider pubertal development when conducting research on youth antisocial behavior. This is important for neurobiological work, but also for behavioral work. There are a number of reasons why pubertal development might not be considered, including problems with self-report measures and the invasiveness and expense of Tanner staging (for discussions of the pros and cons of different pubertal measures, see Dorn et al., 2006, 2019). The development of new technologies over the past 15 years offers substantial progress. Notably, the development of salivary biosciences has made the moment-to-moment assessment of hormone levels feasible logistically, non-invasive, and cost-effective (Granger et al., 2007). Self-report measurement of puberty can reasonably approximate Tanner staging and can be combined with hormonal assessments to yield important data related to pubertal status without an invasive examination (Shirtcliff et al., 2009). Moreover, by considering hormones in addition to the development of secondary sex characteristics, development through the mid-20’s can be quantified. New techniques to assay hormones in hair is also a reliable, cost-effective way of establishing baseline hormones even in menstruating women (Wang et al., 2018). Greater use of these techniques will yield important insights into behavior (Shirtcliff et al., 2009), including antisocial behavior.

Relatedly, better and more consistent use of longitudinal designs will be critical to understand the role of puberty in the development and maintenance of antisocial behavior. It has been noted that many studies that consider puberty examine pubertal timing, an individual’s relative stage of puberty relative to their age, as opposed to pubertal tempo, the pace at which an individual is developing (Mendle et al., 2010). Both timing and tempo will be needed to fully understand pubertal development’s role in antisocial behavior. Longitudinal designs are able to assess both timing and tempo and thus provide a more complete picture of puberty’s role (Beltz et al., 2014; Marceau et al., 2015).

Finally, there needs to be greater collaboration between the legal system and developmental science to develop tools for use in the courtroom. For example, a measure of development/maturity could help inform how juveniles should be considered by the courts. We are unaware of a validated, norm-based measure of maturity that could be easily adapted for use in the courtroom. The tools of psychological practice, currently employed in measures of psychopathology, intellectual functioning, academic achievement, or activities of daily living, could be utilized to provide the courts with an assessment of the maturity of a particular youth relative to their peers. The courts would benefit from an estimate of percentile rank, along with an error rate, from a formal assessment of development and maturity. As noted above, the addition of biomarkers of development (e.g., hormone-based metrics) might eventually prove to add important information in the assessment of maturity (though see section “Reducing Reliance on Age in the Legal System” for hurdles). For these developments to be realized, there must be increased collaboration between developmental researchers and the legal system. Moreover, for test developers to invest in such a measure, it would need to be in demand in the courtroom. We hope that by raising this issue here, the courts, governmental organizations, non-profits, and/or other entities will consider taking steps to partner with developmental scientists and to commit to utilizing evidence-based measures of maturity once they become available.

## Conclusion

Developmental science will best serve the legal system, and most importantly, those individuals being served by the legal system, when the data are as complete and accurate as possible. For scientists studying antisocial behavior, a greater inclusion of puberty and hormonal metrics of development is critical. Both the passage of time and biological changes contribute to development and both will need to be considered to fully understand developmental trajectories to antisocial behavior. For the legal system, the current state of the science indicates that the legal system should be considering variation in development more closely. Development continues long after a youth looks “adult-like” and juveniles should be treated as such for longer than the justice system typically does. Moreover, developmental science suggests that individuals are still maturing, and thus relatively more malleable, in terms of behavior later in life than the justice system currently assumes. Finally, for the legal system to truly be just, human development needs to be more fully considered, and this can only happen if increased collaboration between developmental scientists and legal professionals is leveraged to provide the justice system with the tools that it requires.

## Author Contributions

SW conceptualized and wrote the first draft of the manuscript. SE and EM contributed to the organization, format, and direction of the manuscript. All authors contributed to manuscript revision, read, and approved the submitted version.

## Conflict of Interest

The authors declare that the research was conducted in the absence of any commercial or financial relationships that could be construed as a potential conflict of interest.

## Publisher’s Note

All claims expressed in this article are solely those of the authors and do not necessarily represent those of their affiliated organizations, or those of the publisher, the editors and the reviewers. Any product that may be evaluated in this article, or claim that may be made by its manufacturer, is not guaranteed or endorsed by the publisher.
